# Association of Area-Based Socioeconomic Measures with Tuberculosis Incidence in California

**DOI:** 10.1007/s10903-022-01424-7

**Published:** 2022-11-29

**Authors:** Yasser Bakhsh, Adam Readhead, Jennifer Flood, Pennan Barry

**Affiliations:** 1 Present Address: International Health Regulations Program, Ministry of Health, Riyadh, Saudi Arabia; 2 Tuberculosis Control Branch, California Department of Public Health, 850 Marina Bay Parkway, Richmond, CA USA; 3 Epidemic Intelligence Service, Centers for Disease Control and Prevention, Atlanta, GA USA; 4 Institute for Global Health Sciences, University of California, San Francisco, CA USA

**Keywords:** Tuberculosis, Socio-economic status, Area-based measures

## Abstract

We assessed the association of area-based socio-economic status (SES) measures with tuberculosis (TB) incidence in California. We used TB disease data for 2012–2016 (n = 9901), population estimates, and SES measures to calculate incidence rates, rate ratios, and 95% confidence intervals (95% CI) by SES and birth country. SES was measured by census tract and was categorized by quartiles for education, crowding, and the California Healthy Places Index (HPI)and by specific cutoffs for poverty. The lowest SES areas defined by education, crowding, poverty, and HPI had 39%, 40%, 41%, and 33% of TB cases respectively. SES level was inversely associated with TB incidence across all SES measures and birth countries. TB rates were 3.2 (95% CI 3.0–3.4), 2.1 (95% CI 1.9–2.2), 3.6 (95% CI 3.3–3.8), and 2.0 (95% CI 1.9–2.1) times higher in lowest SES areas vs. highest SES areas as defined by education, crowding, poverty and HPI respectively. Area-based SES measures are associated with TB incidence in California. This information could inform TB prevention efforts in terms of materials, partnerships, and prioritization.

**Supplementary Information:**

The online version contains supplementary material available at 10.1007/s10903-022-01424-7.

## Background

The COVID-19 pandemic has highlighted the disproportionate burden of infectious disease borne by people with lower socio-economic status (SES). Tuberculosis (TB) has long been recognized as a disease that similarly disproportionately affects persons with lower SES globally[1–4]. In high-resource, low-burden settings such as the United States, TB has also been associated with socio-economic status [5–8]. The mechanisms by which SES affects TB are unclear, though reduced access to healthcare, lower quality healthcare, increased comorbidities, and structural racism are thought to increase the risk of TB disease [9–12].

Information on the impact of socio-economic status on TB in California is incomplete. The two studies conducted in California were limited in scope: the first was restricted to pediatric TB and the second focused on case clustering and had limited measures of SES [8, 13]. Furthermore, the recent experience of COVID-19 in California highlighted strong disparities by SES, suggesting that there may be a similar pattern in TB [14].

In California, the epidemiology of TB is dominated by the risk associated with non-U.S. birth [15–17]. In 2020, more than 84% of TB cases occurred in persons born outside the United States and incidence rates among non-U.S.-born were 14 times that of U.S.-born. These disparities are even more pronounced when stratifying by race and ethnicity. The rates among Asian persons and Black persons born outside the U.S. were 50 and 51 times higher than rates among U.S.-born White persons respectively. Furthermore, with the exception of 2016, the percent decline in case burden was less than 1.5% between 2013 and 2019, despite the prior sharp declines in TB incidence in the 1990s and 2000s [15]. In response, California developed a TB elimination plan, which focuses on preventing TB through finding and treating latent TB infection (LTBI) among populations who experience risk for TB [18]. Central to this strategy is a plan to screen nearly 10 million persons born outside the United States who are at elevated risk of TB infection and treat the more than 2 million estimated infected with LTBI, which several models have shown to be an impactful and cost-effective approach [19–24]. Because few additional resources are available for this effort, strategies that can further prioritize TB testing among people born outside the U.S. may be useful and could address disparities.

We investigated whether SES was associated with TB incidence in California overall and whether there were differences in the association by country of birth. We also investigated whether the possible association between SES and TB incidence differed between persons with TB attributed to recent transmission and persons with TB attributed to reactivation of latent infection.

## Methods

We used data from the California TB registry on 10,668 persons with active TB disease reported to the California Department of Public Health during 2012–2016. Residential addresses at first report were geocoded using ArcGIS 10.5 and the Composite Point Address Geolocator with address data provided by ESRI [25]. We were interested in geocoding records to the census tract level because this was the smallest area for which country of birth data, a key risk factor for TB, was available. Census tracts are small, contiguous geographic areas defined by the U.S. Census Bureau which, on average, encompass 4000 people [26]. Of 10,668 records, 591 were excluded: 82 records were marked as homeless and had inadequate address data, 26 were geocoded to census tracts outside California, and 433 lacked sufficient detail to be geocoded to the census tract level, leaving 10,077 records. We further excluded 26 records missing country of birth, and 150 records that were geocoded to census tracts where the population denominator corresponding to the country of birth for the TB case could not be estimated, leaving a total of 9901 records for analysis (Supplement Fig. 1).

**Fig. 1:**
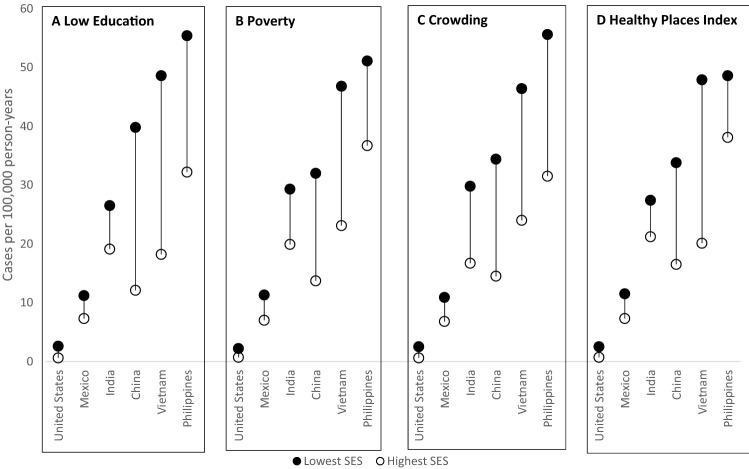
Tuberculosis incidence rates stratified by SES measure, country of birth, and socioeconomic level. The SES measures are: **A** low education (percent of census tract population aged ≥ 25 years with less than high school education), **B** poverty (percent of census tract population below the federal poverty line), **C** crowding (percent of census tract housing units with > 1 person per room), **D** Healthy Places Index (HPI)—California, 2012–2016. Note: Highest and lowest socioeconomic levels are defined by quartiles for education, crowding and health place index and by specific cutoffs for poverty (Supplement Table 1)

**Table 1:** Case counts and incidence rate of TB: overall, by country of birth, and by level of socioeconomic status (SES) measures—California, 2012–2016 (N = 9901)

	Number of cases (%)	Rate per 100,000 person-years (95% CI)	Incidence rate ratio (95% CI)
Overall	9901 (100%)	5.1 (5.0, 5.2)	
Country of birth^a^			
United States	1920 (19%)	1.4 (1.3, 1.4)	Reference
Not United States	7981 (81%)	15.3 (15.0, 15.6)	11.2 (10.7, 11.8)
China	630 (6%)	21.9 (20.2, 23.7)	16.1 (14.7, 17.6)
India	473 (5%)	21.7 (19.8, 23.7)	15.9 (14.4, 17.6)
Mexico	2113 (21%)	10.0 (9.6, 10.4)	7.3 (6.9, 7.8)
Philippines	1801 (18%)	43.1 (41.1, 45.1)	31.7 (29.9, 33.8)
Vietnam	935 (9%)	36.7 (34.4, 39.1)	27.0 (25.0, 29.2)
Low education			
1—Low SES	3907 (39%)	7.9 (7.7, 8.1)	3.2 (3.0, 3.4)
2	2908 (29%)	5.9 (5.7, 6.1)	2.4 (2.2, 2.5)
3	1959 (20%)	4.0 (3.8, 4.2)	1.6 (1.5, 1.7)
4—High SES	1127 (11%)	2.5 (2.4, 2.7)	Reference
Poverty			
1—Low SES	3949 (40%)	6.9 (6.7, 7.1)	2.1 (1.9, 2.2)
2	3120 (32%)	5.1 (4.9, 5.2)	1.5 (1.4, 1.6)
3	1946 (20%)	4.1 (4.0, 4.3)	1.2 (1.2, 1.4)
4—High SES	882 (9%)	3.3 (3.1, 3.6)	Reference
Crowding			
1—Low SES	4095 (41%)	8.3 (8.1, 8.6)	3.6 (3.3, 3.8)
2	2995 (30%)	6.0 (5.8, 6.2)	2.6 (2.4, 2.8)
3	1771 (18%)	3.6 (3.5, 3.8)	1.6 (1.5, 1.7)
4—High SES	1033 (10%)	2.3 (2.2, 2.5)	Reference
Healthy Places Index^b^			
1—Low SES	3278 (33%)	7.0 (6.8, 7.3)	2.0 (1.9, 2.1)
2	2680 (27%)	5.5 (5.3, 5.7)	1.6 (1.5, 1.7)
3	2170 (22%)	4.5 (4.3, 4.6)	1.3 (1.2, 1.3)
4—High SES	1659 (17%)	3.5 (3.4, 3.7)	Reference

^a^“Not United States” includes all non-U.S. countries including the five non-U.S. countries listed

^b^Cases in the Healthy Places Index (HPI) do not add to 9901 because of cases in census tracts that do not have HPI values

Because person-level SES data were not available, we chose an ecological approach, using census tract-level data from the 2012–2016 American Community Survey (ACS) and the California Healthy Places Index (HPI) [27, 28]. From the ACS, we selected three SES measures described in prior literature as being associated with TB incidence: low education, poverty, and crowding [5, 29]. Details of categorization of census tracts by SES measures appear in Supplement Table 1. Low education was defined as the percent of persons, aged 25 years and older, with less than 12th grade education and census tracts were categorized into quartiles by low education, with cut points at 6.0%, 13.6%, 28.0%. Poverty was defined as the percent of population below the federal poverty level. We categorized census tracts into four categories by poverty using a priori cut points of 5%, 10% and 20% following earlier publications and the US census definition of “poverty areas” as those areas where at least 20% of residents were below the Federal poverty level [29, 30]. Crowding was defined as the percent of housing units with more than one person per room and census tracts were categorized in quartiles with cut points at 2.2%, 5.8% and 13.3%. Census tracts were categorized by quartiles using the California Health Places Index, which is a percentile index, so cut points were at 25%, 50% and 75%. The California Healthy Places Index is a composite index of multiple social, demographic and health access variables that has been used widely in California [31].

Using case counts from the TB registry and 5-year average population estimates from ACS data, specifically tables B05002, B05006, S1701, B25014, and S1501, we calculated the incidence rates and 95% confidence intervals for all cases, U.S.-born and non-U.S.-born, and for persons born in each of five non-U.S. countries of interest [27]. Because of concerns regarding sparse data, we focused on U.S.-born persons and persons born in the five countries with the most TB cases in California in this period: China, India, Mexico, Vietnam, and the Philippines. For each SES measure, we also calculated incidence rates by SES category and country of birth. We performed separate analyses stratified by source of TB disease: recent transmission or reactivation of LTBI. We used the Centers for Disease Control and Prevention definition of recent transmission which designates a case as attributable to recent transmission if a plausible source case can be identified [32]. A plausible source case is a person 10-years-old or older who has an infectious form of TB disease with the same genotype, resides within 10 miles of the putative recipient case, and was diagnosed within 2 years before the putative recipient case [32].

We created four negative binomial regression models to examine the relationship between TB incidence rates and census tract-level SES measures. Each of the first three models included an SES measure (low education, poverty or crowding), country of birth, and product term for the SES measure and country of birth. We built an additional model to explore potential confounding of SES measures (Supplement Fig. 2), which included all three SES measures, country of birth, and three product terms, one for each SES measure and country of birth. We assessed multicollinearity between SES measures with the variance inflation factor. SES measures were included in the models in their continuous form and rescaled such that 1 model unit corresponds to a 10% change in the measure. Note that the increases in continuous forms of the SES measures used here correspond to decreases in socio-economic status. For example, an increase in low education variable represents an increase in the percentage of persons aged 25 and older with less than a 12th grade education which corresponds to a reduction in socio-economic status. Results are presented as percent change in TB incidence rate. We evaluated model fit using dispersion statistics and Akaike Information Criterion (AIC) and compared these statistics across models [33]. Also, we investigated the predictive ability of the first three models using a k-fold cross validation. Data were divided into 10 folds. For each fold, the fold data were reserved for testing and the remaining data were used in the model. Mean squared error was calculated for each fold and an overall cross validation metric was calculated by taking the average of the mean squared error for each fold. This analysis was determined by the Centers for Disease Control and Prevention to be a non-research public health activity and did not require human subjects review by an institutional review board.

**Fig. 2:**
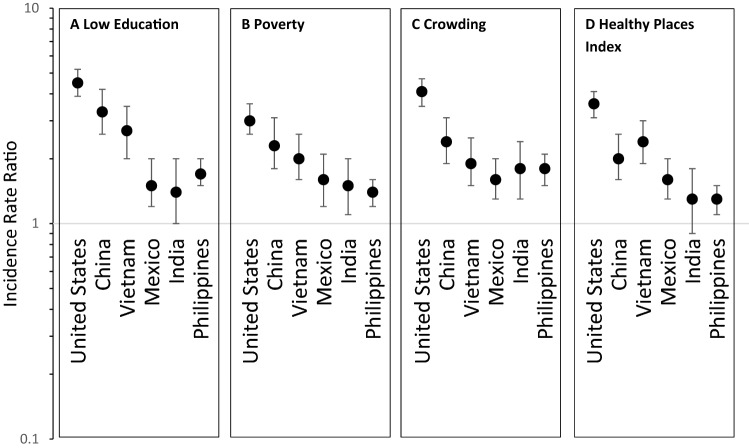
Tuberculosis incidence rate ratios stratified by country of birth comparing lowest to highest socioeconomic levels measured by: **A** low education (percent of census tract population aged ≥ 25 years with less than high school education), **B** poverty (percent of census tract population below the federal poverty line), **C** crowding (percent of census tract housing units with > 1 person per room), and **D** Healthy Places Index (HPI)—California, 2012–2016. Note: Highest and lowest socioeconomic levels are defined by quartiles for education, crowding and health place index and by specific cutoffs for poverty (Supplement Table 1)

## Results

Of 10,668 reports of TB during 2012–2016, 10,077 (94%) had residential addresses in California and had sufficient address data for geocoding (Supplement Fig. 1). After exclusions, 9901 cases (93%) were included for analysis. Of persons included in the analysis, 19% were in U.S.-born person and 81% were in non-U.S.-born persons (Table 1). The overall TB incidence rate was 5.1 cases per 100,000 person-years (95% Confidence Interval (CI) 5.0–5.2) and there were substantial differences in rates by country of birth. Across all measures of SES, areas with low SES had more cases of TB and elevated rates of TB incidence when compared to areas with high SES. Also, for all measures, each increase in SES level (across quartile or category) corresponded to a decrease in TB rate (Table 1). There were, however, some differences in TB incidence by measure. Comparing incidence rates in areas with low SES to those with high SES, the incidence rate ratios were 3.2 (95% CI 3.0–3.4) for low education, 2.1 (95% CI 1.9–2.2) for poverty, 3.6 (95% CI 3.3–3.8) for crowding, and 2.0 (95% CI 1.9–2.1) for the HPI.

There were notable differences in TB incidence by SES when stratified by country of birth (Table 2; Fig. 1). Similar to the unstratified analysis, low SES areas had higher TB incidence rates than in high SES areas across all SES measures and countries of birth. TB incidence rates were highest among Philippines-born persons in low SES areas and lowest among U.S.-born person in high SES areas. Persons born in Mexico had lower rates of TB incidence than other non-U.S. countries examined, but still had elevated incidence among low SES areas compared with high SES areas. Across all SES measures, the incidence rate ratio comparing areas of low and high SES was elevated among U.S.-born persons in comparison to non-U.S.-born persons. Also, rate ratios between low and high SES areas were higher for persons born in China and Vietnam in contrast to ratios for persons born in Mexico and the Philippines (Fig. 2).

**Table 2:** TB incidence rate by level of socioeconomic status (SES) measures and country of birth, California 2012–2016

	Incidence rate per 100 000 person-years (95% CI)				IRR^b^ (95% CI)
	1—Low SES level	2	3	4—High SES level	
Low education					
United States	2.6 (2.5, 2.8)	1.5 (1.4, 1.7)	0.9 (0.8, 1.0)	0.6 (0.5, 0.7)	4.5 (3.9, 5.2)
Not United States^a^	16.5 (16.0, 17.1)	17.1 (16.4, 17.8)	15.1 (14.4, 15.9)	10.1 (9.5, 10.8)	1.6 (1.5, 1.8)
China	39.8 (34.5, 45.9)	26.3 (22.9, 30.2)	17.7 (15.0, 20.9)	12.1 (10.0, 14.7)	3.3 (2.6, 4.2)
India	26.5 (19.0, 36.8)	18.5 (14.6, 23.3)	25.7 (22.3, 29.6)	19.1 (16.5, 22.1)	1.4 (1.0, 2.0)
Mexico	11.2 (10.6, 11.8)	8.8 (8.1, 9.6)	7.1 (6.1, 8.3)	7.3 (5.6, 9.6)	1.5 (1.2, 2.0)
Philippines	55.4 (50.0, 61.3)	47.6 (44.3, 51.2)	37.3 (34.2, 40.7)	32.2 (28.2, 36.9)	1.7 (1.5, 2.0)
Vietnam	48.6 (44.1, 53.6)	38.5 (34.6, 43.0)	26.6 (22.7, 31.1)	18.2 (14.0, 23.7)	2.7 (2.0, 3.5)
Poverty					
United States	2.2 (2.1, 2.4)	1.3 (1.2, 1.4)	0.9 (0.8, 1.0)	0.7 (0.6, 0.9)	3.0 (2.6, 3.6)
Not United States^a^	16.5 (15.9, 17.1)	15.4 (14.8, 16.0)	14.7 (14.0, 15.4)	12.6 (11.7, 13.5)	1.3 (1.2, 1.4)
China	32.0 (27.8, 36.9)	22.2 (19.3, 25.6)	19.6 (16.9, 22.7)	13.7 (10.8, 17.3)	2.3 (1.8, 3.1)
India	29.3 (23.2, 36.9)	20.4 (16.8, 24.7)	21.7 (18.7, 25.2)	19.9 (16.7, 23.6)	1.5 (1.1, 2.0)
Mexico	11.3 (10.7, 11.9)	9.2 (8.5, 10.0)	7.5 (6.5, 8.7)	7.0 (5.4, 9.2)	1.6 (1.2, 2.1)
Philippines	51.1 (46.4, 56.3)	43.1 (39.9, 46.7)	41.2 (37.9, 44.8)	36.7 (32.3, 41.8)	1.4 (1.2, 1.6)
Vietnam	46.8 (42.0, 52.2)	39.6 (35.8, 43.8)	27.5 (23.6, 32.0)	23.1 (18.3, 29.4)	2.0 (1.6, 2.6)
Crowding					
United States	2.5 (2.4, 2.7)	1.6 (1.5, 1.7)	1.0 (0.9, 1.1)	0.6 (0.5, 0.7)	4.1 (3.5, 4.7)
Not United States^a^	17.1 (16.5, 17.7)	16.7 (16.1, 17.4)	13.5 (12.8, 14.2)	10.5 (9.8, 11.3)	1.6 (1.5, 1.8)
China	34.4 (29.9, 39.5)	22.9 (20.1, 26.2)	17.0 (14.3, 20.2)	14.5 (11.7, 17.9)	2.4 (1.9, 3.1)
India	29.8 (24.0, 36.9)	24.0 (20.8, 27.9)	19.6 (16.4, 23.3)	16.7 (13.6, 20.6)	1.8 (1.3, 2.4)
Mexico	10.9 (10.3, 11.5)	9.5 (8.8, 10.4)	8.4 (7.4, 9.6)	6.8 (5.4, 8.6)	1.6 (1.3, 2.0)
Philippines	55.6 (51.2, 60.4)	44.4 (41.0, 48.0)	36.9 (33.5, 40.7)	31.5 (27.5, 36.1)	1.8 (1.5, 2.1)
Vietnam	46.4 (42.3, 50.9)	36.7 (32.7, 41.2)	25.4 (21.2, 30.4)	24.0 (19.2, 30.0)	1.9 (1.5, 2.5)
Healthy Places Index					
United States	2.5 (2.3, 2.6)	1.4 (1.3, 1.5)	1.0 (0.9, 1.1)	0.7 (0.6, 0.8)	3.6 (3.1, 4.1)
Not United States^a^	16.1 (15.5, 16.7)	15.8 (15.1, 16.5)	15.7 (15.0, 16.4)	13.0 (12.4, 13.7)	1.2 (1.2, 1.3)
China	33.8 (28.4, 40.2)	26.0 (22.1, 30.6)	21.3 (18.2, 24.8)	16.5 (14.3, 19.1)	2.0 (1.6, 2.6)
India	27.4 (19.7, 38.2)	20.7 (16.0, 26.7)	21.9 (18.5, 26.0)	21.2 (18.7,24.0)	1.3 (0.9, 1.8)
Mexico	11.5 (10.9, 12.2)	9.1 (8.4, 9.8)	7.6 (6.7, 8.6)	7.3 (5.9, 9.1)	1.6 (1.3, 2.0)
Philippines	48.6 (43.3, 54.5)	48.1 (44.2, 52.5)	40.8 (37.6, 44.2)	38.1 (34.6, 42.1)	1.3 (1.1, 1.5)
Vietnam	47.9 (41.8, 54.9)	43.2 (39.1, 47.7)	33.0 (29.0, 37.6)	20.1 (16.6, 24.4)	2.4 (1.9, 3.0)

^a^“Not United States” includes all non-U.S. countries including the five non-U.S. countries listed

^b^Incidence rate ratio (IRR) compares high SES to low SES

Results of the multivariable analysis show an overall similar pattern to the unstratified analysis and stratified analysis by country of birth (Table 3). In the first three models which included each SES measure separately, the association of low SES with increased TB rate was consistent across all measures except low education and poverty for India, the country with the fewest TB cases in the dataset. Additionally, these models showed the association was strongest among U.S.-born persons. In model four, under specific assumptions implied by the directed acyclic graph (Supplement Table 2), the association of crowding with TB incidence appeared attenuated when adjusted for confounding of poverty and low education on crowding.

**Table 3:** Percent change in TB incidence rate for a 10% decrease in socioeconomic status measure—California, 2012–2016

	Model 1— low education^a^	Model 2—poverty^b^	Model 3—crowding^c^
	Percent change (95% CI)	Percent change (95% CI)	Percent change (95% CI)
United States	38 (35, 42)	41 (36, 47)	53 (47, 60)
China	30 (22, 39)	16 (8, 25)	42 (28, 58)
India	6 (− 4, 17)	10 (− 1, 12)	31 (13, 51)
Mexico	10 (7, 38)	17 (12, 22)	10 (6, 15)
Philippines	16 (11, 21)	13 (7, 19)	25 (17, 33)
Vietnam	21 (15, 28)	21 (12, 31)	27 (17, 38)
Dispersion statistic	1.24	1.20	1.26
Akaike Information Criterion (AIC)	42,074	42,212	42,444
Cross validation metric	0.3260	0.3360	0.3325

^a^Model 1 includes education, country of birth and a product term for country of birth and education

^b^Model 2 includes poverty, country of birth and a product term for country of birth and poverty

^c^Model 3 includes crowding, country of birth and a product term for country of birth and crowding

Although there were fewer cases attributed to recent transmission (1078 cases) than to LTBI reactivation (8819 cases), the association between SES measures and TB incidence was generally stronger among cases attributed to recent transmission (Table 4). However, the four SES measures remained associated with incidence among cases attributed to LTBI reactivation.

**Table 4:** Incidence rate ratios comparing low to high socioeconomic status levels, stratified by place of birth, for TB cases attributed to recent transmission and cases attributed to latent TB infection reactivation—California, 2012–2016

	Recent transmission		LTBI reactivation	
	Number of cases	IRR (95% CI)	Number of cases	IRR (95% CI)
Low education				
United States	403	9.1 (6.1, 13.5)	1517	3.8 (3.3, 4.5)
Not United States	675	3.8 (2.8, 5.3)	7306	1.5 (1.4, 1.7)
China	57	9.9 (3.4, 28.8)	573	3.0 (2.4, 3.9)
India	6	*	467	1.4 (0.9, 2.0)
Mexico	212	2.9 (0.9, 8.9)	1901	1.5 (1.1, 1.9)
Philippines	152	1.6 (0.9, 2.9)	1649	1.7 (1.5, 2.1)
Vietnam	89	16.7 (2.3, 121)	846	2.4 (1.8, 3.2)
Poverty				
United States	403	8.2 (4.9, 13.9)	1516	2.5 (2.1, 3.0)
Not United States	675	2.2 (1.6, 3.0)	7303	1.3 (1.2, 1.4)
China	57	7.6 (1.8, 32.8)	572	2.2 (1.7, 2.9)
India	6	1.3 (0.1, 14.5)	467	1.5 (1.1, 2.0)
Mexico	212	4.7 (1.2, 19.0)	1899	1.5 (1.1, 2.0)
Philippines	152	1.0 (0.6, 1.7)	1649	1.4 (1.2, 1.7)
Vietnam	89	2.1 (0.9, 5.0)	846	2.0 (1.5, 2.7)
Crowding				
United States	403	5.2 (3.7, 7.2)	1515	3.8 (3.2, 4.5)
Not United States	675	3.0 (2.2, 4.1)	7301	1.5 (1.4, 1.7)
China	57	5.2 (1.8, 15.1)	572	2.2 (1.7, 2.9)
India	6	1.9 (0.3, 13.4)	467	1.8 (1.3, 2.4)
Mexico	212	1.4 (0.7, 2.7)	1899	1.6 (1.3, 2.1)
Philippines	152	2.4 (1.3, 4.4)	1649	1.7 (1.5, 2.0)
Vietnam	89	2.4 (1.1, 5.3)	845	1.9 (1.5, 2.4)
Healthy Places Index				
United States	403	6.3 (4.4, 9.0)	1517	3.1 (2.6, 3.6)
Not United States	675	2.4 (1.9, 3.2)	7306	1.2 (1.1, 1.2)
China	57	2.7 (1.2, 6.4)	573	2.0 (1.6, 2.5)
India	6	*	467	1.3 (0.9, 1.9)
Mexico	212	2.0 (0.9, 4.2)	1901	1.5 (1.2, 1.9)
Philippines	152	1.3 (0.8, 2.2)	1649	1.3 (1.1, 1.5)
Vietnam	89	4.5 (1.7, 12.0)	846	2.3 (1.8, 2.9)

High and low socioeconomic levels are defined by quartiles for education, crowding and health place index and by specific cutoffs for poverty (Supplement Table 1)

*IRR could not be estimated because low SES level had zero cases

## Discussion

Our results show inequities by country of birth and SES in the incidence of TB in California. Persons living in census tracts with low SES had higher TB incidence rates than those living in high SES census tracts, whether measured by education level, poverty, crowding or HPI. Our findings align with multiple published studies in the U.S. showing association between SES and TB incidence using area-based SES measures at the census tract, ZIP code and block group level though evidence of this association at larger areas in mixed [5, 6, 17, 34, 35]. Importantly, the association between SES and TB incidence remained strong even when stratifying by country of birth, a major risk factor for TB within the United States. In all country of birth groups, TB rates were higher among persons living in low SES groups. This association was most pronounced among U.S.-born persons, as evidenced by persons in census tracts with the lowest educational attainment having TB rates 4.5 times that of persons in census tracts with the highest educational attainment. Among persons born outside the U.S., notable heterogeneity in rate ratios by country of birth suggest that SES influences TB incidence differently between groups.

Country of birth remained a stronger risk factor for TB than SES but examining the risk by country of birth through the lens of SES differences makes these disparities even more stark. The rate among U.S.-born in the areas with high SES was 0.6–0.7 per 100,000 person-years. The U.S.-born population in these census tracts has reached TB rates consistent with having achieved pre-elimination, which is set at 1 case per 100,000 person-years [5, 36]. In contrast, persons born in the Philippines or Vietnam and living in low SES census tracts had rates of TB incidence ranging from 46 to 56 cases per 100,000 person-years, 65 to 93 times the TB rates among U.S.-born persons in high SES census tracts. These findings highlight the ongoing and significant disparities by SES and country of birth. As with COVID-19 and other infectious diseases, groups experiencing disadvantage are the most affected [5, 14, 37].

These findings reaffirm a focus on TB prevention in non-U.S.-born persons while also providing a rationale for prioritization of TB prevention among non-U.S.-born populations who live in areas with low SES [38]. Such a prioritization may also help reduce disparities in TB rate by country of birth and SES. These results also indicate that strategies to make TB prevention services widely accessible regardless of ability to pay are necessary. Furthermore, these data highlight the potential for public health interventions that address multiple conditions more common in areas of low SES including COVID-19 [14]. Finally, the analysis provides additional support for community-based and place-based interventions, similar to those implemented for LTBI treatment, COVID-19 testing and COVID-19 vaccination among others [39–42].

The association between SES and TB incidence was stronger among cases attributed to recent transmission compared to cases attributed to LTBI reactivation. This is consistent with the idea that poverty and crowding increase the risk of transmitting TB infection. However, SES was also associated with TB incidence among cases attributed to reactivation across all countries of birth. While the classification of cases as recent transmission or reactivation is imperfect, it is helpful to consider several possible explanations for how SES may affect reactivation. One is that non-U.S.-born persons living in low SES areas in California lived in low SES areas in their country of birth and therefore experienced higher risk for exposure before arriving in the United States. Additionally, immigrants have a higher rate of TB soon after arrival in the U.S. and new arrivers may also live in areas with low SES [43]. Another possible explanation is that persons living in low SES areas have less access to healthcare, lower quality healthcare, more comorbidities, and that one or more of these factors increases the likelihood of reactivation of TB disease [9–11].

The finding that TB incidence is influenced by SES to a greater degree in U.S.-born persons than in non-U.S.-born persons could be related to the higher proportion of U.S.-born cases attributed to recent transmission (21%) compared to the proportion of non-U.S.-born cases attributed to recent transmission (8%). This finding is also consistent with the fact that U.S.-born persons with TB are more likely to experience homelessness or incarceration, or have a history of substance misuse, compared to non-U.S.-born persons with TB [44]. Although U.S.-born persons in low SES areas had higher TB incidence rates than U.S.-born persons in higher SES areas had, these rates were still less than half the incidence rate in non-U.S.-born persons in high SES areas. This finding argues against prioritizing routine screening for TB among U.S.-born persons even in low SES areas.

Additional interventions among immigrants prior to arrival in the United States could also address disparity by country of birth. A pilot study of such a program has yielded promising results, but would not reach immigrants already living in California [45]. Current programs conducted outside the U.S. are primarily designed to prevent importation of active TB disease among applicants for permanent U.S. residency [46].

Results of the multivariable models were consistent with the stratified analysis. Building models allowed us to use continuous SES data and to account for statistical interaction between country of birth and SES measures. However, the impact of SES on TB incidence clearly is multidimensional and may act through multiple mechanisms and pathways. Because of this, regression models that include multiple SES measures should be interpreted with caution [47].

Authors of a 2011 report recommended that educational attainment and crowding be added to the national surveillance of TB to better track the effect of social determinants of health on the disease [48]. Findings here show that both measures are associated with increased TB incidence. While national TB surveillance has not adopted these measures, California started collecting educational attainment as part of TB surveillance in 2022. In stratified analysis, education showed the strongest association between SES and TB incidence among persons born in China, Philippines, United States, and Vietnam.

Our study had several strengths, including the use of census tract-level data instead of ZIP code tabulation area and incorporation of recent transmission estimates. However, our analysis was also subject to limitations. The main limitation to our approach is that it is an ecologic analysis. Area-based SES measures may not adequately represent individual SES, though studies have shown reasonable agreement in urban areas [49, 50]. Additionally, we were unable to account for other confounders of SES and TB incidence such as age and occupation because there were no additional stratifications of country-of-birth population estimates available at the census tract level. We did not directly account for dependent outcomes, that is transmission between cases, but we did perform separate analyses for cases attributed to recent transmission and reactivation. Our analysis was limited to persons born in six countries. Association between SES and TB incidence may not be generalizable to persons born in other countries. Also, we used the Census Bureau definition of poverty, which uses similar income thresholds for all states, and might not apply well to California where the cost of living is generally high [51]. In multivariable models, although we considered multiple plausible causal pathways, the true pathway is unknown, and causal inferences should be made with caution. The analysis of recent transmission relies on a definition that has been validated in the field but has limitations including reliance on standard genotyping methods with imperfect discriminatory power as well as less granular spatial and temporal data which could result in misclassification of recent transmission [32]. Because this is an analysis of observational data, unknown and unmeasured confounders could affect our results. Lastly, confidence intervals calculated in the unstratified and stratified analyses use the Poisson distribution and are likely to under-estimate variability because the outcome is not independent.

## Conclusion

Demonstrating significant health inequities, SES measured at the census tract level is associated with TB incidence rate. Although person-level SES data are unavailable in TB surveillance data, use of area-based SES measures in analyses of TB surveillance data can yield important information that could inform TB prevention efforts. Our findings reaffirm the value of focusing TB screening based on country of birth, because non-U.S.-born persons showed considerably higher incidence and overall burden compared to U.S.-born persons living in low SES areas. However, SES could be used to tailor TB prevention activities among non-U.S. born persons. For example, local TB control programs and health systems could promote TB screening among non-U.S.-born persons by educating providers serving populations with low SES, such as providers working in Federally Qualified Health Centers or serving Medicaid patients. When designing community TB screening campaigns, efforts could be focused on reaching persons with low SES among the census tracts with higher numbers of persons born outside the United States. Campaigns could seek partnerships with organizations who focus on persons with low SES as well as tailor materials such that they are effective in reaching populations experiencing disadvantage. These results should also be a reminder that TB prevention programs should address barriers faced by persons in low SES areas such as cost and difficulty accessing TB care.

## Supplementary Information

Below is the link to the electronic supplementary material.

Supplementary material 1 (DOCX 64.3 kb)

## Abbreviations

TB: Tuberculosis
SES: Socio-economic status
CI: Confidence interval
ACS: American Community Survey
LTBI: Latent tuberculosis infection
IRR: Incidence rate ratio
HPI: Healthy Places Index
