# Supplementary material for: Association of Area-Based Socioeconomic Measures with Tuberculosis Incidence in California
Source: J Immigr Minor Health. 2022 Nov 29;25(3):643–52. doi: 10.1007/s10903-022-01424-7 (PMC9707420; doi:10.1007/s10903-022-01424-7)
Supplement: Supplementary file 1 — Supplementary material 1 (DOCX 64.3 kb) [file 10903_2022_1424_MOESM1_ESM.docx]

Total TB cases extracted from state registry with report date in 2012–2016

10,668

Exclude:

- 82 marked as homeless
- 26 geocoded to census tracts outside California
- 483 lacking details in address for geocoding

10,077

Exclude:

- 26 missing country of birth
- 150 corresponding with zero estimated population for country of birth in census tract

9,901

Supplement Figure 1: Flowchart of analytic population

Poverty

TB incidence rate

Crowding

Low Education

Country of birth

Supplement Figure 2: a plausible directed acyclic graph showing assumption made to explain the association of socioeconomic measures with TB incidence rate

Supplement Table 1: SES category definitions and the number and percentage of census tracts in each category

|  | **Cut-points (%)** | **Census-tracts in category (%)** |
| --- | --- | --- |
| **Poverty: percent of population below Federal poverty limit** |  |  |
| 1 – Low SES | 20–100 | 2457 (30.8) |
| 2 | 10–19.9 | 2480 (31.1) |
| 3 | 5–9.9 | 1917 (24) |
| 4 – High SES | 0–4.9 | 1203 (14.2) |
| Missing |  | 72 (0.9) |
|  |  |  |
| **Crowding: percent of housing units with more than one person per room** |  |  |
| 1 – Low SES | 13.3–100 | 2001 (25.1) |
| 2 | 5.8–13.2 | 1994 (25) |
| 3 | 2.2–5.8 | 1986 (24.9) |
| 4 – High SES | 0–2.1 | 2000 (25.1) |
| Missing |  | 76 (0.9) |
|  |  |  |
| **Low Education: percent of persons, age 25 and older, with less than 12^th^ grade education** |  |  |
| 1 – Low SES | 28–100 | 2013 (25.1) |
| 2 | 13.6–27.9 | 2015 (25.2) |
| 3 | 6–13.5 | 2018 (25.2) |
| 4 – High SES | 0–5.9 | 1963 (24.5) |
| Missing |  | 48 (0.6) |
| **Healthy Places Index: percentile of census tracts with lower HPI score** |  |  |
| 1 – Low SES | 0-24.9 | 1948 (24.2) |
| 2 | 25.0-49.9 | 1948 (24.2) |
| 3 | 50.0–74.9 | 1948 (24.2) |
| 4 – High SES | 75-100 | 1948 (24.2) |
| Missing |  | 264 (3.3) |

Supplement Table 2: Regression analysis results for percent change in TB incidence rate comparing census tracts with 10% difference in socioeconomic status measures — California, 2012–2016*

|  | Low Education | Poverty | Crowding |
| --- | --- | --- | --- |
|  | Percent change (95% CI) | Percent change (95% CI) | Percent change (95% CI) |
| **United States** | 29 (22, 36) | 15 (9, 20) | 1 (-7, 9) |
| **China** | 31 (17, 46) | -3 (-12, 7) | 2 (-15, 22) |
| **India** | -11 (-22, 3) | 4 (-7, 17) | 42 (16, 74) |
| **Mexico** | 6 (1, 12) | 13 (7, 19) | -4 (-10, 3) |
| **Philippines** | 8 (1, 18) | -2 (-9, 6) | 15 (4, 27) |
| **Vietnam** | 20 (8, 34) | 1 (-9, 6) | 1 (-13, 17) |

*Model includes education, poverty, crowding, country of birth, and product terms for country of birth and each SES measure
